# qPCR based mRNA quality score show intact mRNA after heat stabilization

**DOI:** 10.1016/j.bdq.2016.01.002

**Published:** 2016-02-10

**Authors:** Oskar Karlsson, Lova Segerström, Robert Sjöback, Ingrid Nylander, Mats Borén

**Affiliations:** a Department of Clinical Neuroscience, Karolinska Institutet, The Center for Molecular Medicine, Stockholm, Sweden; b Department of Pharmaceutical Biosciences/Pharmacology/Neuropharmacology, Addiction & Behavior, Uppsala University, Uppsala, Sweden; c TATAA Biocenter, Göteborg, Sweden; d Denator AB, Uppsala, Sweden

**Keywords:** Heat stabilization, mRNA quality, RT-qPCR, Post-sampling change, Sample preparation

## Abstract

Analysis of multiple analytes from biological samples can be challenging as different analytes require different preservation measures. Heat induced enzymatic inactivation is an efficient way to preserve proteins and their modifications in biological samples but RNA quality, as measured by RIN value, has been a concern in such samples. Here, we investigate the effect of heat stabilization compared with standard snap freezing on RNA quality using two RNA extraction protocols, QiaZol with and without urea pre-solubilization, and two RNA quality measurements: RIN value, as defined by the Agilent Bioanalyzer, and an alternative qPCR based method. DNA extraction from heat stabilized brain samples was also examined. The snap frozen samples had RIN values about 1 unit higher than heat stabilized samples for the direct QiaZol extraction but equal with stabilized samples using urea pre-solubilization. qPCR based RNA quality measurement showed no difference in quality between snap frozen and heat inactivated samples. The probable explanation for this discrepancy is that the RIN value is an indirect measure based on rRNA, while the qPCR score is based on actual measurement of mRNA quality. The DNA yield from heat stabilized brain tissue samples was significantly increased, compared to the snap frozen tissue, without any effects on purity or quality. Hence, heat stabilization of tissues opens up the possibility for a two step preservation protocol, where proteins and their modifications can be preserved in the first heat based step, while in a second step, using standard RNA preservation strategies, mRNA be preserved. This collection strategy will enable biobanking of samples where the ultimate analysis is not determined without loss of sample quality.

## 1 Introduction

The removal of a biological sample from its *in vivo* environment initiates a series of signaling cascades as the cells in the sample adapt to their new environment [14]. Analyte levels, *e.g.,* peptides, protein phosphorylation, lipids and metabolites, will start to change from their actual *in vivo* levels, distorting analysis results and making interpretation of analytical results difficult or even impossible [5], [15]. A range of preservation techniques are used to deal with post-sampling change but they are usually optimized to preserve a specific analyte or class of analytes while not preserving other analytes. This approach is generally satisfactory as long as only analytes from a single analyte class are analysed. However, analysis of multiple analytes from precious biological samples can be challenging as different analytes require different preservation measures. Analysis of both nucleic acids, *e.g.,* RNA and DNA, as well as protein phosphorylation from a single sample is an example of such a situation. This is particularly challenging when selecting collection strategies for biobanking sample where the ultimate analysis is not known at the time of sampling. Classical RNA preservation strategies, *e.g.,* RNA Later, while preserving RNA quality do not prevent the more rapid changes to protein phosphorylation that occur post-sampling. Heat induced enzymatic inactivation on the other hand have been shown to be an efficient way to preserve protein phosphorylation but RNA quality has been a concern in such samples [7], [12]. RNA quality as measured using electrophoretic analysis of RNA, *e.g.,* RIN value using an Agilent Bioanalyzer assay, has indicated a reduction in quality in samples after heat denaturation and a further reduction in RIN value when heat stabilized samples are incubated at room temperature indicating that RNases remain active after heat denaturation [12]. Heat stabilization can thus not be used alone as a way to preserve RNA but enable extraction of RNA as a secondary analyte from samples where proteins are the primary analyte of interest. The RIN value is primarily based on rRNA rather than mRNA, which is the RNA type typically analyzed, and give only a surrogate quality score for mRNA [13]. The ideal RNA quality score would rather be based on direct quantification of mRNA. Various approaches for using qPCR to measure mRNA quality have been proposed, *e.g.,* the use of a ratio Cq from 2 shorter qPCR assays [10] or comparing delta Cq of multiple 3′ amplicons of a single assay [16]. Another qPCR based mRNA quality score has been developed by TATAA Biocenter [1], [6]. Their qPCR based RNA quality score measure the difference in amplification rate between a long and a short qPCR amplicon of a number of mRNA transcripts, selected based on their post-sampling degradation characteristics.

The aim of the present study was to investigate the effect of heat based sample stabilization through protein denaturation done using the Stabilizor system (Denator, Sweden) on RNA quality using Bioanalyzer (RIN value) and TATAAs RT-qPCR (mRNA based) quality scoring. If mRNA can be shown not to be degraded by heat stabilization in the Stabilizor system it would enable analysis of the primary analyte, *e.g.,* protein phosphorylation, as well as mRNA as a secondary analyte both with high quality. DNA extraction from heat stabilized brain samples was also examined to investigate the possibility to also analyze DNA from heat stabilized samples.

## 2 Material and methods

### 2.1 Animal housing and sample collection

Male adolescent Wistar rats, 36 in total, (RccHan:WIST, Harlan Laboratories, The Netherlands) were acclimatized one week prior to the experiments. They were housed three per cage with food and water *ad libitum*. Animal rooms were temperature- and humidity-controlled and kept on a reversed 12 h light/dark cycle with lights off at 6:00 a.m. The animal experiments were approved by the regional ethics committee and were in accordance with the Swedish Animal Protection Legislation.

**Fig. 1:**
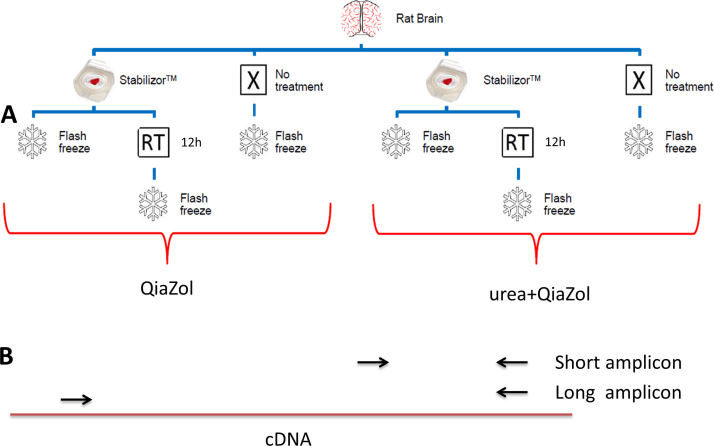
Panel A: experimental layout. Sections of rat brain were either snap frozen or heat stabilized using the stabilizor T1. The heat stabilized samples were then either directly snap frozen or further incubated for 12 h at room temperature to determine stabilizing effect on RNA by heat stabilization. The samples were collected in duplicates to enable comparative extraction with two extraction protocols, either directly with QiaZol or with a pre-solubilization step with 8 M urea prior to QiaZol extraction. Each sample group consisted of four biological replicates, *N* = 4. Panel B: schematics of qPCR based RNA quality scoring. Two overlapping qPCR reactions, one short and one long, is amplified from the same cDNA. The difference in Ct between the long and the short will depend on cDNA fragmentation and becomes an indicator of RNA quality.

The rats were sacrificed one at a time using CO_2_ and decapitated when euthanized. The brain was removed from the scull right after decapitation and two adjacent sagital sections of 1 mm thickness was cut from the posterior part of the cerebrum for the RNA experiments. From eight animals, one section was snap frozen directly while the other was heat stabilized with the Stabilizor™ System (Denator, Sweden) using the Auto Fresh mode prior to snap freezing. Four additional animals were dissected in a similar way but both sections were heat stabilized individually and then incubated for 12 h at room temperature prior to being frozen, see Fig. 1 for an experimental outline. For the DNA experiments cerebellum was removed from 24 additional animals and subsequently treated using six different protocols (*n* = 4 per protocol). Two groups were either instantly dissected from fresh tissue, or dissected after heat stabilization of the intact brain. The brains from the third and fourth groups of animals were kept at room temperature for 60 and 300 min, respectively, to allow for post-sampling changes to appear before the samples were snap frozen. The brains from the fifth and sixth group were removal from the scull and heat stabilization as whole brains of the brain instantly dissected after and left at room temperature for 60 min and 300 min, respectively, before the samples were snap frozen. All tissue samples were stored at −80 °C prior to tissue extraction.

### 2.2 RNA extraction

Total RNA was extracted from the samples as follows: The samples were homogenized while frozen using the Tissuelyser II (Qiagen) and extraction buffer added directly to the frozen powder. Extraction was done using QiaZol and Qiagen RNeasy Mini Kit either according to Qiagen manual for RNeasy Mini Kit or by including a urea pre-solubilization step. One set, constituting an identical half of the samples were pre-solubilization with 10 volumes, *i.e.,* 10 μl per 1 mg of tissue, of urea buffer (8 M urea, 0.5% SDS, 10 mM Tris, pH 8, 5 mM EDTA, 0.25 M NaCl). This was followed by the transfer of 400 μl urea buffer sample extract to a new tube and 600 μl of QiaZol was added to lyse the samples. The other half set of samples was extracted directly in 1 ml QiaZol. An extra step of centrifugation (1 min, 6000 × *g*) after homogenization was added to all samples due to foaming of the samples.

### 2.3 RNA yield, purity, quality and normalization

The concentration and purity (A260nm/A280nm and A260nm/A230nm) of the 24 extracted RNA samples was measured on DropSense96 (Trinean). The quality of the RNA was measured on BioAnalyzer (Agilent Technologies) to assign RIN-values to each RNA extract. For further analysis the samples were normalized to 100 ng/μl using the DropSense96 value.

### 2.4 cDNA synthesis and qPCR based long and short RNA quality assays

cDNA was synthesized from each sample using TATAA Grandscript cDNA synthesis kit #A103 (TATAA Biocenter AB) with a mix of random and poly-dT primers with 500 ng of total RNA as starting material. qPCR was performed with TATAA SYBR^®^ GrandMaster Mix # TA01 (TATAA Biocenter AB). Reverse transcription was performed in single measurements in 10 μl reaction volume on CFX96 (Bio-Rad Laboratories, Inc.) with the following program, 5 min at 22 °C, 30 min at 42 °C, 5 min at 85 °C, hold at 4 °C. Negative controls were included during extraction and a NoRT control was added to check for DNA contamination. RNA quality was checked using three qPCR based long and short RNA quality assays: two based on mRNA transcripts (B2 M and mtCO1) and one based on the 18S rRNA transcript (for validation data, please see Ref. [2]). qPCR was performed on cDNA equivalent to 20 ng RNA per sample with TATAA SYBR^®^ GrandMaster Mix # TA01 (TATAA Biocenter, Sweden). All samples including extraction negatives, NoRT, gDNA and NTC were run in duplicates in 10 μl reactions on LightCycler480 platform (Roche, Basel, Switzerland). All pipetting was performed by a pipetting robot (EpMotion 5070, Eppendorf, Germany). The temperature program, melting for 60 s at 95 °C followed by 45 cycles of 20 s at 60 °C alternating with 20 s at 72 °C, was used and detection was performed in the SYBR channel.

### 2.5 DNA extraction

DNA extraction was done using GeneElute™ Mammalian Genomic DNA miniprep kit (Sigma–Aldrich). In brief, brain samples were homogenized manually with a micropestle in 1.5 ml Eppendorf tubes together with lysis buffer containing Proteinase K. The samples were incubated at 55 °C for 4 h to allow for tissue digestion. RNase was added to allow for extraction of RNA-free genomic DNA. DNA isolation was done using spin columns according to the manual for the Kit. The DNA was eluted in 100 μl of elution solution. To increase the elution efficiency, the tubes were incubated for 5 min with the elution solution prior to centrifugation to ensure equilibration. After centrifugation the collected elution solution was pipetted back on the binding column and the elution procedure was repeated once.

### 2.6 DNA yield, purity and quality

The concentration and purity (A260nm/A280nm and A260nm/A230nm) of the 24 extracted DNA samples was measured on a Nanodrop (Thermo Scientific). The quality of the DNA was further evaluated with gel electrophoresis. Equal amounts of DNA (100 ng) were loaded on a 1% agarose gel and subjected to electrophoresis at 70 V for 90 min. DNA bands were visualized with UV light after ethidium bromide staining.

## 3 Results and discussions

The RNA yield, purity and quality has been measured in RNA extracts from heat stabilized and snap frozen brain tissue samples with the aim of investigating the effect of heat denaturation on two different RNA quality measurements, either Bioanalyzer (primarily rRNA based) or qPCR (mRNA based).

### 3.1 RNA yield and purity

**Fig. 2:**
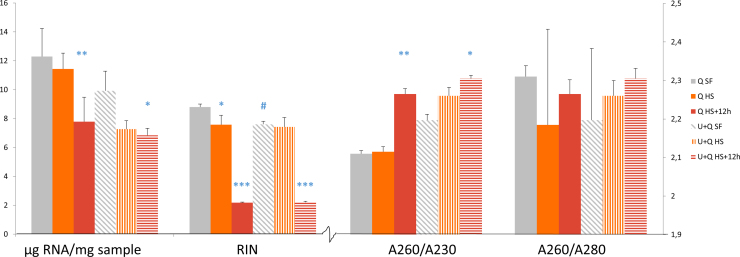
Yield, quality and purity of extracted RNA (mean ± SD). Three different treatment groups, *n* = 4, (SF-snap frozen, HS-heat stabilized, HS + 12 h-heat stabilized with 12 h incubation at room temperature after heat stabilization), extracted with two different protocols (Q-QiaZol buffer and U + Q-Urea pre-solubilization prior to QiaZol extraction) were compared. μg RNA/mg tissue-yield of RNA from samples (left scale), RIN-RNA quality score as measured using the bioanalyzer (left scale), A260/A280-assesment of protein contamination (right scale), A260/A230-assesment of contaminants such as guanidine thiocynate and carbohydrates (right scale). *p < 0.05, **p < 0.01, ***p < 0.001 compared with snap frozen samples (SF) within respective extraction protocol, ^#^*p* < 0.05, compared to corresponding group in non-urea pre-solubilized samples (ANOVA followed by Tukey HSD test; *n* = 4).

RNA was extracted using two different protocols and yield and purity as measured using A260nm/A280nm ratio, see Fig. 2. Direct extraction in QiaZol yielded the highest RNA levels; with equal levels for both snap frozen and heat stabilized samples. Heat stabilized samples with 12 h of room temperature incubation after stabilization showed lower levels. The second extraction protocol included a pre-solubilization in urea prior to QiaZol. Urea pre-solubilization resulted in lower yields especially with heat stabilized samples. The A260nm/A280nm ratio and the A260nm/A230nm ratio, are high, >2, for all samples indicating that the contamination of the RNA with proteins and other contaminants such as phenol, guanidine thiocyanate and carbohydrates is low. This shows that the extraction works well regardless of pre-treatment and buffer as measured with the inherent limitations of A260nm/A280nm ratio [3], [4], [8], [17].

### 3.2 RNA quality as measured by RIN value

The RIN value, as determined using the Bioanalyzer, is primarily based on rRNA and functions as a surrogate quality marker for mRNA. Snap frozen samples pre-solubilized in urea prior to QiaZol had significantly lower RIN values compared to snap frozen samples directly extracted in QiaZol (Fig. 2). The RIN values for heat stabilized samples were equal for the two extraction procedures. The snap frozen samples had RIN values about 1 unit higher than heat stabilized samples for the direct QiaZol extraction but equal with stabilized samples using the urea pre-solubilization. The urea pre-solubilization step was included as an attempt to gently break up ribosomal protein-RNA complexes giving better rRNA quality and RIN value. Although RIN value levels were equal for heat stabilized and snap frozen samples with the urea pre-solubilization, the levels are lower than with the direct QiaZol extraction indicating that pre-solubilization with urea is not an efficient way of extracting higher levels of intact rRNA from heat stabilized samples but instead lowers the extraction efficiency of snap frozen samples resulting in lower RIN score. It has been speculated that the difference in rRNA based quality scores, *e.g.,* RIN value, for heat stabilized *versus* snap frozen samples could be attributed to the association of rRNA with proteins, enzymes and extensive chemical reactants and that rapid heating may cause rRNA-protein complexes to cleave and degrade the rRNA [9].

### 3.3 RNA quality as measured by qPCR based markers

As most RNA analyses are based on mRNA, it would be more correct to base the RNA quality assessment on mRNA rather than rRNA. qPCR amplification is quantitative and well suited to measure levels of individual mRNA transcripts that can be used to asses RNA quality. Various approaches for using qPCR to measure mRNA quality have been proposed, e.g., the use of a ratio Cq from 2 shorter qPCR assays [10] or comparing delta Cq of multiple 3′ amplicons of a single assay [16]. The qPCR based quality measure used in this work is based on the difference in Cq between two qPCR reactions, a long and a short amplicon that shares one primer, from the same transcript, see schematic in Fig. 1. The RNA quality score is the difference in Cq between the two reactions, less difference higher quality. Three different qPCR based assays, based on two different mRNAs and 18s rRNA, showing different sensitivity to degradation has been used to measure RNA quality, as defined in [1], [2].

**Fig. 3:**
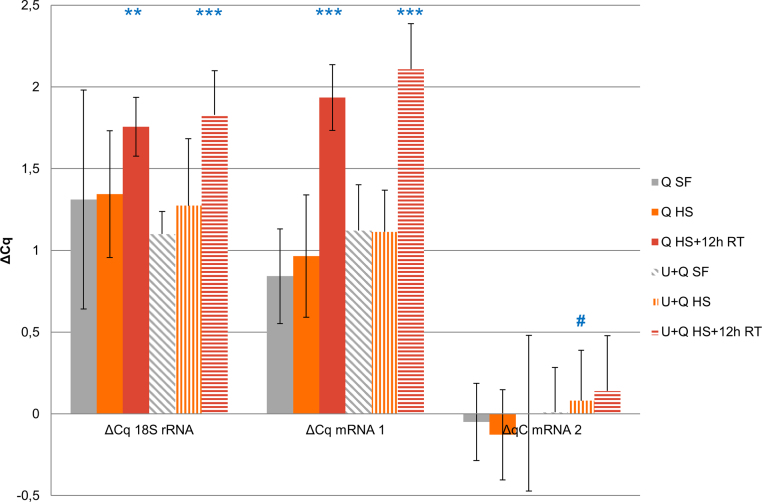
qPCR based RNA quality determination (mean ± SD). Three different treatment groups, *n* = 4, (SF-snap frozen, HS-heat stabilized, HS + 12h-heat stabilized with 12 h incubation at room temperature after heat stabilization), extracted with two different protocols (Q-QiaZol buffer and U + Q-Urea pre-solubilization prior to QiaZol extraction) were compared. Difference in Cq, ΔCq, between long and short amplification frames, as measured for three different RNA targets indicate mRNA quality. **p < 0.01, ***p < 0.001 compared with snap frozen samples (SF) within respective extraction protocol, ^#^*p *< 0.05, compared to corresponding group in non-urea pre-solubilized samples (ANOVA followed by Tukey HSD test; *n* = 4).

The qRCR mRNA quality score was very close for all three qPCR markers tested for both snap frozen and heat stabilized samples regardless of extraction method (Fig. 3). This shows that mRNA is not degraded by heat stabilization in the Stabilizor system and that high quality mRNA can be extracted equally well from snap frozen samples as well as samples stabilized by heat denaturation of enzymes. The quality score was markedly increased, indicating lower quality, for the heat stabilized samples with 12 h room temperature incubation (Fig. 3). This is in close correlation to the decrease in RIN value for these samples suggesting continued RNA degradation after heat stabilization (Fig. 2). These results indicate ongoing RNase activity probably due to refolding of RNases and resulting gradual loss of RNA quality in heat stabilized samples stored at room temperature [11]. The ability to refold after heat denaturation has been attributed to small protein size and several disulfide bonds which hold the protein together while denatured [11]. This imply that heat stabilized samples intended for extraction of both proteins and nucleic acids should be taken care of directly after heat stabilization either by freezing or a second RNA preservation step, *e.g.,* RNAlater, to enable extraction of both high quality proteins and RNA.

### 3.4 DNA yield, purity and quality

**Fig. 4:**
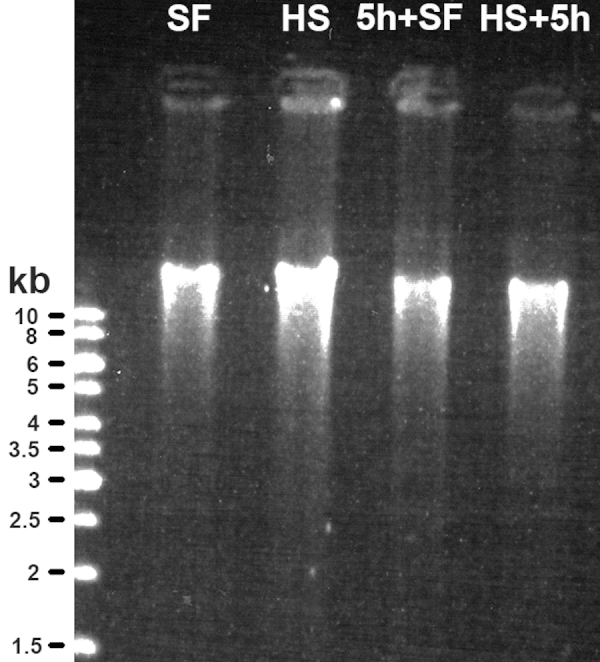
The gel electrophoresis showed that the extracted DNA was of high molecular weight and no clear DNA fragmentation. (SF-snap frozen, HS-heat stabilized, 5 h SF -left in room temperature for 5 h to allow for post-sampling changes to appear, HS + 5 h -heat stabilized with incubation at room temperature after heat stabilization for 5 h).

The DNA yield or the A260nm/A280nm and A260nm/A230nm ratios were not affected in samples that were kept at room temperature for 60 or 300 min, to allow for post-sampling changes to appear, compared to samples that were directly snap frozen (Table 1). The DNA yield from heat stabilized brain tissue samples was increased, compared to the snap frozen tissue, without any effects on purity or quality (Table 1). This shows that DNA is not degraded by heat stabilization in the Stabilizor system and that high quality DNA may be extracted from samples stabilized by protein heat denaturation with even higher yield than from snap frozen samples. It should be noted that the GeneElute™ Mammalian Genomic DNA miniprep Kit (Sigma–Aldrich) gave a relatively high yield variation that increased with incubation time at room temperature in this experiment (Table 1). Despite this heat stabilized samples with 60 and 300 min at room temperature gave significantly higher DNA yields (Table 1). The gel electrophoresis revealed that the extracted DNA was of high molecular weight with no clear DNA fragmentation (Fig. 4). This confirms the general belief that DNA is relatively resistant to post-sampling degradation.

## 4 Conclusion

Analysis of multiple analytes from precious biological samples can be challenging as different analytes require different preservation measures. Although heat inactivation does not preserve RNA from further post-sampling degradation the results in this study show that heat stabilization does not directly affect mRNA quality when using the mRNA based qPCR quality measure and confirms the previously reported decreased in quality when using the primarily rRNA based RIN quality value. Further studies with other RNA quality scores and more samples would be required to understand the apparent difference in RNA quality between the two quality metrics and RNA sub-classes. However, this finding opens up the possibility of a combination protocol where proteins and their modifications can be preserved by heat stabilization while retaining high quality DNA and mRNA. To ensure long-term stability of mRNA, standard RNA preservation strategies*, e.g.,* freezing or RNA Later should be added in a second step. This will enable multiple analyses of DNA, mRNA and protein phosphorylation with high quality from the same sample.

## Conflict of interests

M.B. works for Denator, the manufacturer of the Stabilizor system. R.S. works for TATAA Biocenter, the CRO that invented and performed the qPCR based mRNA quality scoring used in this study.

## Figures and Tables

**Table 1:** Yield and purity of extracted DNA (mean ± SD). Six different treatment groups, (SF-snap frozen, 60 min SF and 300 min SF-left in room temperature for the indicated time to allow for post-sampling changes to appear, HS-heat stabilized, HS + 60 min and HS + 300 min-heat stabilized with incubation at room temperature after heat stabilization for the indicated time). A260/A280-assesment of protein contamination, A260/A230-assesment of contaminants such as guanidine thiocynate and carbohydrates. **p < 0.01 compared with snap frozen samples (SF), ^##^*p* < 0.01, compared to corresponding time in room temperature for snap frozen samples (ANOVA followed by Tukey HSD test test; *n* = 4).

Group	μg DNA/mg sample	260/280	260/230
SF	892 ± 132	1.91 ± 0.01	2.30 ± 0.06
60 min SF	827 ± 247	1.90 ± 0.01	2.26 ± 0.10
300 min SF	733 ± 324	1.91 ± 0.00	2.58 ± 0.53
HS	1367 ± 320	1.90 ± 0.01	2.23 ± 0.03
HS + 60 min	2502 ± 1057**^,##^	1.90 ± 0.03	2.11 ± 0.16
HS + 300 min	2510 ± 871**^,##^	1.90 ± 0.00	2.22 ± 0.08
